# Violent Video Game Exposure and Problem Behaviors among Children and Adolescents: The Mediating Role of Deviant Peer Affiliation for Gender and Grade Differences

**DOI:** 10.3390/ijerph192215400

**Published:** 2022-11-21

**Authors:** Mingchen Wei, Yanling Liu, Shuai Chen

**Affiliations:** Research Center of Mental Health Education, Faculty of Psychology, Southwest University, Chongqing 400715, China

**Keywords:** violent video game exposure, deviant peer affiliation, problem behavior, child adolescents, gender differences, grade differences

## Abstract

Based on problem behavior theory, a mediation model for gender and grade differences is explored in this study. The study examined gender and grade differences in the effects of violent video games and deviant peer affiliation on problem behaviors among children and adolescents. A total of 2118 children and adolescents from four primary and middle schools in China (M age = 13.08, SD = 2.17) were surveyed using an anonymous questionnaire on basic information, exposure to violent video games, deviant peer affiliation, and problem behaviors. The results showed that exposure to violent video games significantly positively predicted problem behaviors, and deviant peer affiliation played a mediating role. Significant gender and grade differences were found in the mediating effect. This finding helps understand the individual differences in the influencing factors of problem behaviors. Further, it has important implications for interventions to reduce problem behaviors among children and adolescents.

## 1. Introduction

Problem behavior (PB) is one of the most prevalent and persistent forms of maladjustment among children and adolescents [1]. It refers to abnormal behaviors that occur in individuals that hinder their social adjustment, involving both their own emotional abnormalities and behaviors that negatively affect others and society [2]. Further, it can be divided into internalizing and externalizing problems. Research has shown that PBs among children and adolescents are often strongly associated with lower academic performance [3], substance addiction [4], adjustment difficulties [5], and even lead to delinquent behavior [6]. Moreover, this adverse effect often persists into adulthood [7]. Therefore, it is necessary to study the factors influencing children and adolescents’ PBs to reduce the occurrence of their PBs and promote the healthy development of primary and middle school students.

Problem behavior theory (PBT) suggests that PBs do not occur randomly among children and adolescents but result from a combination of risk and protective factors. Protective factors reduce the likelihood of PBs by providing prosocial behavior patterns and individual or social regulation of PBs. Contrastingly, risk factors increase the likelihood of PBs by providing PB patterns, greater exposure or involvement in PBs, and increased individual susceptibility to PBs [8,9,10].

Since the beginning of the 21st century, with the rapid development of the Internet, video games have been an important leisure and entertainment tool for children and adolescents. Violent video games, as a popular game genre, are vital for the development of children and adolescents. Based on PBT, violent video games serve as a virtual external environment that can influence the production and dissolution of PBs among children and adolescents. Scholars have investigated the effects of violent video games on children and adolescents. Through multiple meta-analyses, they established that violent video games significantly adversely affected aggressive behavior, aggressive affect, violent desensitization, and mental health [11]. Moreover, few studies have found that deviant peer affiliation (DPA) may mediate the relationship between risk factors and PBs. For example, Bao et al. (2015) found that school climate indirectly predicts juvenile criminal behavior through adverse peer interactions [12]. Sun and Sun (2021) suggested that peer influence may mediate the relationship between violent video games and PBs [13]. Violent video games and peers are critical environmental factors that affect children’s and adolescents’ PBs.

Notably, in addition to risk and protective factors, PBT emphasizes the possible differences in PB by gender and age [14]. Previous studies have found that boys have more PBs than girls [15], and adolescent PBs increase with age. Further, Moffitt (2003) argues that adolescent antisocial behavior peaks at approximately 17 years of age [16].

Through literature review, we found that research on how violent video games affect PBs is limited, with inadequate discussion of social context and participants who are middle school or college students. Simultaneously, fewer studies involve middle and elementary school students. Therefore, based on a literature review and PBT, this study investigated the effects of violent video game exposure (VVGE) on children and adolescents’ PBs, including the mediating effect of DPA, with Chinese middle and elementary school students as participants. Moreover, we focused on the differences in the relationship between VVGE, DPA, and PB among children and adolescents of different genders and grades.

### 1.1. Violent Video Game Exposure and Problem Behavior

Most studies show that violent video games significantly increase PB in players. Regarding violent content, some meta-analyses have found that violent video games increase players’ aggressive cognition, emotion, and behavior and decrease players’ empathy, which negatively impacts players’ social behavior [17,18]. Concerning game frequency, studies have found that long-term video game exposure has adverse effects on adolescents’ emotions and social relations and increases the risk of anxiety, mood disorders, and social adjustment difficulties [19,20]. Further, attention difficulties and hyperactivity disorder are significantly related to game exposure [21]. Some empirical studies have also shown that VVGE is associated substantially with PBs such as aggressive behavior [22] and emotional problems, including anxiety and depression [23,24], and even criminal behavior [25]. Based on the literature reviewed above, we propose the following hypothesis:

**Hypothesis** **1.** *VVGE positively correlates with PBs among children and adolescents*.

### 1.2. Deviant Peer Affiliation as a Mediator

DPA refers to associating with peers who violate school rules and regulations, social ethics, and legal behaviors [26]. Evidence shows that DPA is significantly associated with individual PBs [27,28]. The General Aggression Model is often used to explain and predict the paths and ways in which violent video games lead to increased aggression. Recent revisions to the General Aggression Model theory have highlighted the role of peer groups in the association between violent video games and their adverse effects [29]. This suggests that DPA may be an important mediating variable between VVGE and PBs among children and adolescents. As children and adolescents join the school and adopt it as their main life scene, they rely more on their peers (classmates). At this stage, children and adolescents have a strong need for a sense of belonging to the group, and peers are vital in the adolescent development stage, especially in the transition to middle school. According to the “interpersonal similarity principle”, children and adolescents seek peers with the same or similar behaviors to establish friendships based on their own characteristics. Children and adolescents who play violent games are more likely to choose peers with similar behavioral characteristics to develop friendships with them [30]. Studies have found that VVGE affects the social behavior of players and those associated with them, such as increased aggression by players and their peers [31,32]. Some studies on actual violence have also found that exposure to community violence may increase the likelihood of adolescents becoming friends with immoral peers [33]. To avoid deviating from the social context of peer norms or peer group roles, adolescents may conform to their peers’ interests, hobbies, and behaviors based on adverse peer pressure [34] and then develop more problematic behaviors.

Similarly, DPA also affects individual behavior; it is one of the common risk factors for externalization problems (EP) [35]. Studies have found that children or adolescents with aggressive friends are more likely to have social adjustment problems [30] and that DPA is also commonly associated with skipping class, smoking, violence, or aggression [36,37]. Regarding internalization problems (IP), some researchers believe that the quality of friendships adolescents establish in DPA is low, and the relationship between groups is chaotic. The effort and return between them is inadequate; therefore, it is difficult to obtain necessary emotional support, which is more likely to lead to IPs, such as emotional disorders [38,39]. Based on the literature reviewed above, we propose the following hypothesis:

**Hypothesis** **2.** *Deviant peer affiliation significantly mediates the relationship between exposure to violent games and PBs among children and adolescents*.

### 1.3. Gender and Grade Differences

Although gender and grade differences in PBs have been widely acknowledged and discussed, few studies explore the factors influencing PBs among children and adolescents of different genders and grades. Based on PBT, as external environmental factors, VVGE and DPA may have significant gender and grade differences in their relationship with PB.

First, concerning gender, previous studies have shown that boys indulge in violent video games more than girls do. They have a lower level of guilt when exposed to violent video games [40]. Most researchers also believe that boys are more likely to be influenced by immoral peers and that they have various internal and external problems [41,42]. However, some research results show that girls are more likely to be influenced by immoral peers in drinking behavior [43] and depression or anxiety [44]. Second, at grade level, studies have found that playing time decreases with age [45], and that game preferences change. The susceptibility or resistance of individuals to peer influence also presents different characteristics at various ages. For example, studies have found that the tendency of individual behavior to be susceptible to peer influence seems to appear in early adolescence and reaches its peak at approximately 14 years of age [46]. This study focuses on the mediating effect of adverse peer interactions between VVGE and problem behavior. Based on this, we propose the following hypothesis:

**Hypothesis** **3.** *Significant gender and grade differences exist in the relationships between VVGE, DPA, and PBs among children and adolescents*.

### 1.4. Research Purpose

**Figure 1:**
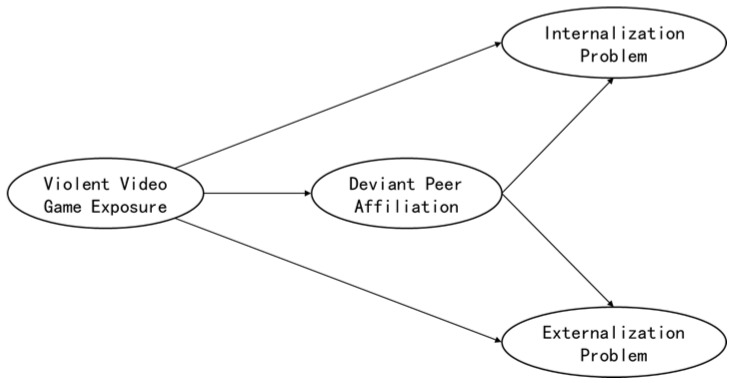
Hypothesized model of violent video games affecting problem behavior.

In summary, this study intends to use Chinese middle and elementary schools as the research object and propose a mediating model (Figure 1) to explore the mediating effect on the relationship between VVGE and PBs among children and adolescents. Based on PBT, the differences in gender and grade were investigated.

## 2. Materials and Methods

### 2.1. Participants

This study used the cluster sampling method to randomly select 2191 students from the fourth grade of elementary school to the first grade of senior high school from four elementary and secondary schools in a city in southwest China. The questionnaire was screened according to the principles, whether the answer was complete, whether there was a regular answer, and whether there was a contradiction. After screening questionnaires, 2118 valid questionnaires were obtained, with an effective rate of 96.67%. The participants were aged between 9 and 17 years, with an average age of 13.08 ± 2.17 years.

Monte Carlo power analysis for indirect effects was adopted. Based on the effect sizes of previous research [47,48], the effects size was set for a (r = 0.10), b (r_externalization problem_ = 0.20, r_internalization problem_ = 0.10), and c (r_externalization problem_ = 0.20, r_internalization problem_ = 0.10) path. Consequently, recruiting a total of 1100 (externalization problem) and 1300 (internalization problem) participants would lead to the power of 90 percent for the indirect effects (i.e., a · b) at the *p* < 0.05 level with 20,000 Monte Carlo replications. Thus, the sample recruited for this study (n = 2118) was more than sufficient to provide adequate statistical power (>95 percent) to detect small indirect effects.

### 2.2. Measures

#### 2.2.1. Violent Video Game Exposure Questionnaire

This questionnaire was compiled by [49] and revised by Chinese scholars, and is widely used [50,51]. It asked the participants to list three violent video games they had most recently encountered and to rate the frequency and violence of each game. Using a 5-point score, the degree of exposure to violent video games = amount of exposure = ∑ [violent content × frequency of using games]/3; the higher the score, the more exposure to violent video games. The violent video game exposure questionnaire has been shown to be both reliable and valid [52]. In this study, Cronbach’s α was 0.87.

#### 2.2.2. Deviant Peer Affiliation Questionnaire

The deviant peer affiliation questionnaire [53,54] contained eight questions that involved destructive behaviors, such as smoking, drinking, theft, Internet addiction, truancy, or misbehavior. A 5-point score from 1 “none” to 5 “all” indicated the number of friends who showed one of the eight destructive behaviors in the recent year. Considering the average score, the higher the score, the more undesirable the peer behavior. The questionnaire has been shown to be reliable and valid [55,56]. In this study, Cronbach’s α was 0.78.

#### 2.2.3. Strengths and Difficulties Questionnaire (Student Version)

This scale was developed by Goodman R. [57,58] and revised by Du [59]. The scale comprises five factors; because this study focused on PBs, the difficult part of the questionnaire was used. This comprised four factors: emotional symptoms, conduct problems, hyperactivity and attention disorders, and peer relationships, and was divided into internalization (emotional symptoms, peer relationships) and externalization problems (conduct problems, hyperactivity, and attention disorders) using Goodman’s suggestion. A “0–2” scale was used, ranging from “does not meet” to “fully meets”. In previous studies, this questionnaire showed good reliability [60,61]. In this study, Cronbach’s α coefficient for the difficult part of the questionnaire was 0.76.

### 2.3. Procedure and Data Analysis

Participants voluntarily completed the questionnaire survey while at school during a specified class period lasting 20 min. SPSS 26.0 was applied to obtain descriptive statistics, Pearson correlations, and difference test. Data may be missing on some variables because participants left an answer blank for 0.28% to 0.94% of the items across the total sample. We used participants’ average scores to replace such information. Then, Amos 24 was applied to test the mediating effect of deviant peer affiliation using the structural equation model, and the differences in gender and grade in the mediating effect of deviant peer affiliation utilizing the method of group analysis.

### 2.4. Common Method Deviation

In this study, variable data collection adopted a self-report method. Based on Zhou and Long’s (2004) suggestion, the Harman single-factor test was adopted to test the common method deviation [62]. The results showed nine factors with eigenvalues > 1, and the variance explained by the first principal factor was 17.35%, which was less than the critical standard of 40%. It can be concluded that there were no common methodological deviations in this study.

## 3. Results

### 3.1. Descriptive Statistics

Table 1 contains the basic information on all subjects. Table 2 contains the correlation, mean, and standard deviation for study variables. Table 3 contains the differences in the research variables in gender and grade.

The results in Table 2 show that there is a significant positive correlation between VVGE and IP and EP, indicating that the more VVGE, the higher the IPs and EPs levels among children and adolescents. Further, there is a significant positive correlation between VVGE and DPA, indicating that the more VVGE, the more DPA among children and adolescents. DPA positively correlates with the IP and EP of children and adolescents, indicating that the more DPA children and adolescents have, the higher their IPs and EPs levels are.

Difference test showed significant differences in VVGE and DPA by gender and grade level—boys had higher levels of VVGE and DPA than girls did. There were substantial differences in IP by gender and non-significant differences by grade level, and girls had higher levels of IPs than boys did. Significant differences existed in EP by grade level and non-significant differences by gender. Children and adolescents in higher grades had more EPs (Table 3).

### 3.2. Mediating Effect of Deviant Peer Affiliation between Violent Video Game Exposure and Problem Behaviors

A structural equation model was constructed using Amos24 to examine the mediating effect of DPA between VVGE and PB. According to the different test results, gender and grade were included in the mediation effect test as control variables.

Based on the mediation effect test process suggested by [63], an unmediated model with VVGE as the independent variable and IP and EP as the dependent variables is constructed. The results showed that the unmediated model fit well (χ^2^/*df* = 11.15, RMSEA = 0.07, CFI = 0.96, TLI = 0.93, GFI = 0.98, IFI = 0.96, SRMR = 0.04), and VVGE positively predicted IPs (*β* = 0.15, *p* < 0.001) and EPs (*β* = 0.24, *p* < 0.001).

A mediating model was constructed using DPA as the mediating variable. The results showed that the mediation model fit well (χ^2^/*df* = 10.33, RMSEA = 0.07, CFI = 0.95, TLI = 0.92, GFI = 0.97, IFI = 0.95, and SRMR = 0.05). The test results for each coefficient showed that VVGE significantly positively predicted DPA (*β* = 0.24, *p* < 0.001), and DPA significantly positively predicted IP. After adding DPA as a mediating variable, the predictive effect of VVGE on IP weakened (*β* = 0.10, *p* < 0.001). DPA positively predicted EP (*β* = 0.38, *p* < 0.001), while the predictive effect of VVGE on EP was weakened (*β* = 0.17, *p* < 0.001), indicating that DPA partially mediated the relationship between VVGE and IP and EP. Bootstrap (sampling times: 5000) was used further to test DPA’s mediating effect (Table 4). The results showed that the mediating effect of DPA between VVGE and IP was 0.06, and 0.09 between VVGE and EP.

### 3.3. Cross-Group Comparison of Mediating Effects of Deviant Peer Affiliation between Violent Game Exposure and Problem Behavior

#### 3.3.1. Gender Difference Analysis

**Figure 2:**
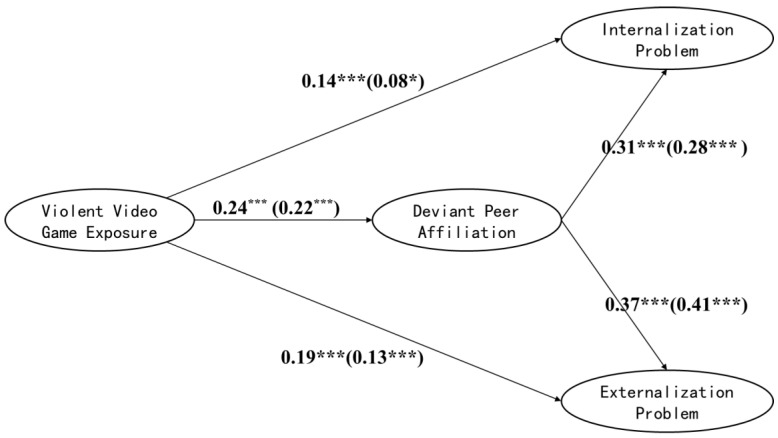
Gender differences in mediating role of deviant peer affiliation. Note: The path coefficient is male outside the brackets and female inside the brackets; the load coefficient between each latent variable and its index, the residual error and error of all variables, and the control variable coefficient are omitted to simplify the model. * *p* < 0.05, *** *p* < 0.001.

A structural equation model was used to investigate gender differences in the mediating effect of DPA between VVGE and PB, and the model included grade as a control variable. First, a model test was conducted on male and female students, and the results showed that the model fit was good in the two samples, which could be compared across groups (Table 5). Next, the unconstrained Model M_0_ was constructed on this basis (the male and female students had the same shape, and the path coefficient was freely estimated). The measurement weights Model M_1_ was constructed based on model M_0_ (the factor loading of latent variables in the two groups of models was restricted to remain unchanged across the groups). The two models fit well. The model comparison showed a significant difference between M_0_ and M_1_ [Δχ^2^ = 20.55, *p* < 0.001], indicating that the model measurement coefficients of the male and female groups had cross-group inequalities. Further tests found cross-group differences in the loading coefficients of factors 1 and 2 on VVGE and the loading coefficients of peer barriers on IP. Therefore, the above coefficients were freely estimated to establish Model M_2_ with equal measurement weights across groups. Finally, the structural weights Model M_3_ is constructed based on M_2_ (the path coefficients in the two groups of models are restricted to remain unchanged across groups). The results showed that both M_2_ and M_3_ models fit well, and the model comparison revealed a significant difference between M_2_ and M_3_ [Δχ^2^ = 57.14, *p* < 0.001]. Further tests revealed that the path coefficients of DPA on IP and EP differed across the groups (Table 5, Figure 2). The predictive effect of DPA on IP in boys (*β* = 0.31, *p* < 0.001) was significantly higher than that in girls (*β* = 0.28, *p* < 0.001). However, the predictive effect of DPA on EP was significantly lower in boys (*β* = 0.37, *p* < 0.001) than in girls (*β* = 0.41, *p* < 0.001).

#### 3.3.2. Grade Difference Test

**Figure 3:**
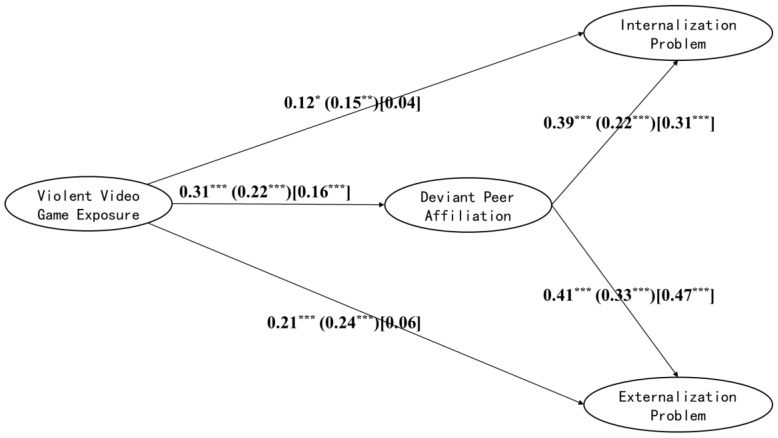
Grade differences in mediating role of deviant peer affiliation. Note: Path coefficients outside parentheses are for elementary school, inside parentheses are for junior high school, and inside square brackets are for senior high school. The load coefficient between each latent variable and its index, the residual error and error of all variables, and the control variable coefficient are omitted to simplify the model. * *p* < 0.05, ** *p* < 0.01, *** *p* < 0.001.

This study investigated the grade difference of the mediating role of DPA in VVGE and PB used for the cross-group comparison of the structural equation model and considered gender as a control variable in the model. The participants were divided into elementary school, junior high school, and senior high school according to their grades, and the grade differences in the intermediary model were investigated. First, the samples were divided into school sections for the model test. The results showed that the models of the three samples fit well and could be compared across groups (Table 6). Next, the unconstrained model M_4_ (three groups of models have the same shape, and the path coefficient can be estimated freely) was built. Further, the measurement coefficient Model M_5_ (limiting the factor load of latent variables in three groups of models to be constant across groups) was built. Both models fit well, and the comparison of the models showed a significant difference between Models M_4_ and M_5_ [Δχ^2^ = 45.66, *p* < 0.001], indicating that the measurement coefficients of the three groups of models were unequal across groups. Further tests showed cross-group differences in the load coefficient of factor 1 and factor 2 on VVGE, the load coefficient of factor 2 on DPA, and the load coefficient of peer obstacles on IP. Therefore, the above coefficients were estimated freely, and Model M_6_ with equal measurement weights s across groups was established. Finally, a structural weights Model M_7_ was built based on M_6_ (the path coefficient among the three models is limited to be unchanged across groups). The results showed that the M_6_ and M_7_ models fit well, and the comparison indicated that M_6_ and M_7_ had significant differences [Δχ^2^ = 110.07, *p* < 0.001]. Further examination showed that cross-group differences existed between the path coefficients of DPA on IP and EP and VVGE on EP (Table 6, Figure 3). For instance, the predictive effect of DPA on IP was significantly higher at the elementary school level (*β* = 0.39, *p* < 0.001) than at the senior high school level (*β* = 0.31, *p* < 0.001), and significantly higher at the senior high school level than at the junior high school level (*β* = 0.22, *p* < 0.001). Further, the predictive effect of DPA on EP was significantly higher at the elementary level (*β* = 0.41, *p* < 0.001) than at the junior high school level (*β* = 0.33, *p* < 0.001), but considerably lower than that at the senior high school level (*β* = 0.47, *p* < 0.001). The direct effect of VVGE on EP was significantly lower at the senior high school level (*β* = 0.06, *p* = 0.217) than at the elementary school level (*β* = 0.21, *p* < 0.001) and junior high school (*β* = 0.24, *p* < 0.001).

## 4. Discussion

Childhood and adolescence are crucial periods of rapid individual development and tremendous change. This study was conducted with elementary, junior high, and senior high school students, focusing on the mechanisms of VVGE and DPA on PBs of children and adolescents and examining the differences by gender and grade.

First, the findings showed that VVGE positively and significantly predicted PB in children and adolescents, validating Research Hypothesis 1, which is consistent with previous research [19,20,21]. Regarding IPs, Kuss and Griffiths (2012) argue that the emergence of IPs may result from escapism and that players are attracted to games to escape from real-world problems [64]. Nonetheless, too much exposure is unhelpful and may exacerbate players’ emotional problems, such as anxiety and depression. Concerning EPs, violent video games are often fast-paced and offer frequent rewards or novel and enjoyable stimuli, which may contribute to the attention problems of child/adolescent players. According to the social learning theory, individuals can acquire undesirable behaviors in two ways: first, through observational learning, in which individuals receive unwanted behaviors by observing or imitating others’ undesirable behaviors. The other way is direct learning, in which individuals acquire undesirable behaviors through personal participation. During the game, child and adolescent players can develop unwanted behaviors by observing and imitating game non-player characters or directly manipulating game characters, which may lead to more EPs being exhibited by children and adolescents in real life.

Second, DPA mediated the relationship between VVGE and IP and EP, validating research Hypothesis 2 that VVGE affects PB by increasing children’s adolescents’ DPA. Games have a significant impact on children’s and adolescents’ peer interaction as a way of maintaining friendship. According to social network theory [65], individuals who are chronically exposed to violent games may be increasingly exposed to undesirable peers through a process of “selection”. The “selection” process refers to children and adolescents actively choosing peers with similar behaviors as their peers. Children and adolescents chronically exposed to violent video games tend to have more IPs or EPs. They may voluntarily join peers identical to them through a selection process based on similarity. The influence of undesirable peers on children and adolescents’ PB results from the interaction between children and adolescents and undesirable peers. This interaction is two-way, including the “socialization” process and the “selection” process. The process of “socialization” refers to children and adolescents making friends with peers who exhibit PB; they may develop similar behaviors under the influence of peer pressure and other factors. These two processes interact; children and adolescents who play games select peers with behavioral problems, and interactions with undesirable peers increase their own problem behaviors [66]. Simultaneously, friendships with undesirable peers tend to be unstable, and interactions with them tend to increase traditional peer rejection, which also increases the risk of IPs and EPs in children and adolescents.

Finally, the study’s results found that the effects of DPA on IP and EP showed significant gender and grade differences, partially validating research Hypothesis 3. Regarding gender, boys’ IPs were more likely to be influenced by DPA. Generally, boys are less flexible and lack cognitive and emotional coping skills and regulation when dealing with interpersonal relationships than girls [34], which may lead to more psychological distress in boys when dealing with DPA. Girls’ EPs are more likely to be influenced by DPA. On the one hand, girls are typically more precocious than boys. They are more likely to be exposed to older mixed-sex groups. Further, adolescent girls are more sensitive to social appraisal concerns and more dependent on intimate relationships as a source of self-evaluation and self-worth [67]. A qualitative study found that college girls’ drinking behavior is more often driven by pressure to impress their male peers [68], suggesting that girls are more likely to engage in more EPs out of the need for approval and influence from undesirable peers. On the other hand, boys have a greater tendency toward EPs than girls, and their EPs are more likely to be influenced by a combination of other factors, such as hormones and personality [69]. Concerning grade differences, elementary school students’ IPs were affected more by DPA. Early adolescence is a critical transition period for individual development, during which essential changes in the individual are often accompanied by changes in the social environment. Simultaneously, individuals become more independent in the face of their parents, where dependence on parents is replaced by reliance on peers, and they are more susceptible to peer influence [70,71]. Additionally, children in elementary school tend to show a one-off imbalance in their psychological development due to their young age. Simultaneously, they have low self-centeredness and self-control; they are more susceptible to peer pressure and more likely to develop IPs. The EPs of senior high school students are affected more by DPA. It has been found that undesirable peer influences play an important role in developing PB in middle and late adolescence [27]. In senior high school, peer interaction has become an important way for senior high school students to meet their social needs and plays an important role in learning and life. Simultaneously, the character and hobbies of peers are gradually becoming essential criteria for choosing friends, and individuals who interact with undesirable peers often tend to misbehave. Aggression and popularity are often intertwined during interactions with undesirable peers [72]. Therefore, senior high school students in unwanted peer groups may adopt destructive behaviors to maintain friendships.

This study is an effort to explore the role of DPA between VVGE and PB, as well as gender and grade differences in a large sample (more than 2000 people), covering children and adolescents from different grades in elementary school, junior high school, and senior high school. In the term of theoretical aspect, this study explores the role of social factors in the relationship between VVGE and children and adolescents’ PBs and made clear the differences in both gender and grade, which deepens our understanding of the impact of VVGE on children and adolescents’ PBs and helps to explain its potential impact mechanism. In practice, this can also bring some thinking for the education of children and adolescents. As one of the common entertainment tools for children and adolescents, violent video games have a certain negative impact on children and adolescents’ social interaction and PBs. At the same time, the DPA of children and adolescents significantly affects their IPs and EPs. Therefore, in terms of family, parents should supervise children’s network use, cultivate children’s healthy network use habits, and avoid excessive addiction to video games. At the same time, they should also pay attention to children’s social interaction, guide children to establish healthy and positive peer groups, and reduce communication with bad peers. In school, first of all, teachers and mental health experts should pay attention to network security, to educate and guide children and adolescents to correctly understand the violent factors in video games. Secondly, we should pay attention to the harm of students’ bad companions and help students to establish a healthy and positive circle of friends. In addition, parents and schools should pay attention to the differences among primary, middle, and high school students in educating and guiding children and adolescents. In the social aspect, we can create a healthy network environment for children and adolescents by improving the game classification system and combating the illegal dissemination of harmful information.

Although the current study yielded important and practical findings on the targeted intervention and guidance, several limitations should be noted. First, this study is a cross-sectional study, which is not plausible to make a causal inference. In the future, longitudinal studies can be applied to clarify the causal relationship between variables. Second, all variables in the study are self-reported and may be affected by common method bias. Therefore, a more comprehensive data collection method should be adopted. Thirdly, video games are one of the common entertainment tools for children and adolescents and are regarded as an important factor affecting the psychosocial development of children and adolescents. This study only focuses on the impact of VVGE on children and adolescents’ PBs. Future research can further discuss the differences between different video game content. Finally, future researchers should consider more potential impact factors. In this study, we found that there are significant gender and age differences in the mediating role of DPA between violent video game exposure and problem behaviors, and the development of peer relationships in children and adolescents is affected by the individual growth environment (such as parent–child relationship, school atmosphere) [12,73]. Therefore, examining environmental factors such as family and school helps us understand the role of deviant peer affiliation.

## 5. Conclusions

In sum, this study found that VVGE can affect the social interaction of children and adolescents, thereby increasing their PBs, and this effect has gender and age differences. In terms of gender, although boys generally show more EPs in their daily lives, their IPs are more susceptible to peer influence, while girls are the opposite. In terms of age, the influence of peers presents different characteristics in different age groups. The IP of primary school students is more susceptible to DPA, while the EP of high school students is more susceptible to DPA.

## Figures and Tables

**Table 1:** Basic information.

		Number	Percentage (%)
Gender	Male	1052	49.7
	Female	1066	50.3
Grade	Elementary school	621	29.3
	Middle school	706	33.3
	High school	791	37.3

**Table 2:** Correlation.

	M ± SD	1	2	3	4
1. VVGE	4.44 ± 3.98	1			
2. DPA	1.25 ± 0.37	0.19 ***	1		
3. IP	0.55 ± 0.31	0.07 **	0.20 ***	1	
4. EP	0.52 ± 0.30	0.19 ***	0.32 ***	0.50 ***	1

Note: ** *p* < 0.01, *** *p* < 0.001.

**Table 3:** Difference test.

Variable		VVGE	DPA	IP	EP
Gender	Male	5.71 ± 4.25	1.27 ± 0.40	0.52 ± 0.30	0.52 ± 0.30
	Female	3.18 ± 3.25	1.22 ± 0.33	0.58 ± 0.32	0.51 ± 0.30
	*t*	15.40 ***	2.56 *	−4.02 ***	1.14
Grade	Elementary school	3.89 ± 3.81	1.15 ± 0.27	0.57 ± 0.32	0.47 ± 0.31
	Middle school	4.23 ± 3.93	1.21 ± 0.34	0.54 ± 0.32	0.52 ± 0.30
	High school	5.06 ± 4.08	1.35 ± 0.43	0.55 ± 0.30	0.55 ± 0.28
	*F*	16.60 ***	62.91 ***	1.83	15.72 ***

Note: * *p* < 0.05, *** *p* < 0.001.

**Table 4:** Breakdown table of the total effect, direct effect, and mediating effect.

Effect	Path	*β*	*SE*	Bootstrap 95%CI		Effect Proportion
				Upper Limit	Lower Limit	
Total effect	VVGE→IP	0.17	0.03	0.11	0.23	
	VVGE→EP	0.26	0.03	0.19	0.33	
Direct effect	VVGE→IP	0.10	0.03	0.05	0.16	62.50%
	VVGE→EP	0.17	0.03	0.10	0.24	65.38%
Indirect effect	VVGE→DPA→IP	0.06	0.01	0.05	0.09	37.50%
	VVGE→DPA→EP	0.09	0.01	0.07	0.12	34.62%

**Table 5:** Fitting index of multi-cohort model for multi-cohort analysis of gender difference.

Model	χ^2^/df	CFI	GFI	IFI	TLI	RMSEA	SRMR
M _male_	4.52	0.97	0.98	0.97	0.94	0.06	0.05
M _female_	7.15	0.95	0.96	0.95	0.92	0.08	0.06
M_0_	5.97	0.96	0.97	0.96	0.93	0.05	0.05
M_1_	5.81	0.95	0.97	0.95	0.93	0.05	0.05
M_2_	5.79	0.96	0.97	0.96	0.93	0.05	0.05
M_3_	5.77	0.95	0.96	0.95	0.93	0.05	0.05

Note: M _male_ and M _female_ were male and female models.

**Table 6:** Fitting index of multi-cohort model for multi-cohort analysis of grade difference.

Model	χ^2^/df	CFI	GFI	IFI	TLI	RMSEA	SRMR
M _elementary school_	1.10	1.00	0.99	1.00	1.00	0.01	0.02
M _junior high school_	2.04	0.99	0.98	0.99	0.98	0.04	0.03
M _senior high school_	2.89	0.98	0.98	0.98	0.96	0.05	0.03
M_4_	2.01	0.99	0.99	0.99	0.98	0.02	0.02
M_5_	2.29	0.98	0.98	0.98	0.97	0.03	0.04
M_6_	1.99	0.99	0.98	0.99	0.98	0.02	0.03
M_7_	2.66	0.97	0.97	0.97	0.96	0.03	0.08

Note: M _elementary school_, M _junior high school_, and M _senior high school_ are models with different school sections.

## Data Availability

Data are available from the corresponding author upon reasonable request.
